# Supplementary material for: Obesogenic Lifestyle and Its Influence on Adiposity in Children and Adolescents, Evidence from Mexico
Source: Nutrients. 2020 Mar 19;12(3):819. doi: 10.3390/nu12030819 (PMC7146202; doi:10.3390/nu12030819)
Supplement: Supplementary file 1 [file nutrients-12-00819-s001.pdf]

**Suplementary Table S1.** Goldberg cut-off limits to classify 1 day dietary recalls based in the ratio of EI:BMR.

| Age (years) | Sex | PAL | Under-report | Plausible report | Over-report |
|---|---|---|---|---|---|
| 3 to < 10 y | Female | low | EI:BMR ≤ 0.79 | 0.79 < EI:BMR < 2.44 | 2.44 ≤ EI:BMR |
| | | moderate | EI:BMR ≤ 0.94 | 0.94 < EI:BMR < 2.89 | 2.89 ≤ EI:BMR |
| | | vigorous | EI:BMR ≤ 1.09 | 1.09 < EI:BMR < 3.36 | 3.36 ≤ EI:BMR |
| | Male | low | EI:BMR ≤ 0.79 | 0.79 < EI:BMR < 2.44 | 2.44 ≤ EI:BMR |
| | | moderate | EI:BMR ≤ 0.93 | 0.93 < EI:BMR < 2.88 | 2.88 ≤ EI:BMR |
| | | vigorous | EI:BMR ≤ 1.08 | 1.08 < EI:BMR < 3.33 | 3.33 ≤ EI:BMR |
| 10 to 17 y | Female | low | EI:BMR ≤ 0.86 | 0.86 < EI:BMR < 2.64 | 2.64 ≤ EI:BMR |
| | | moderate | EI:BMR ≤ 1.0 | 1.0 < EI:BMR < 3.08 | 3.08 ≤ EI:BMR |
| | | vigorous | EI:BMR ≤ 1.15 | 1.15 < EI:BMR < 3.55 | 3.55 ≤ EI:BMR |
| | Male | low | EI:BMR ≤ 0.88 | 0.88 < EI:BMR < 2.7 | 2.7 ≤ EI:BMR |
| | | moderate | EI:BMR ≤ 1.02 | 1.02 < EI:BMR < 3.16 | 3.16 ≤ EI:BMR |
| | | vigorous | EI:BMR ≤ 1.19 | 1.19 < EI:BMR < 3.67 | 3.67 ≤ EI:BMR |

PAL: physical activity level, EI: reported energy intake, BMR: basal metabolic rate calcutaed from Schofield equations.

**Supplementary Table S2.** Misreporting bias of the total sample and by BMI categories.

| BMI category | Under-report | Plausible report | Over-report | *Energy expenditure (kcal) | Energy Intake reported (kcal) | Difference | % adequacy |
|---|---|---|---|---|---|---|---|
| Underweight | 0 | 43 (67%) | 21 (33%) | 1854.6 ± 477 | 2522.5 ± 682 | -667.9 ± 708 | 136 ± 41 |
| Healthy weight | 18 (2%) | 725 (80%) | 168 (18%) | 2130.7 ± 583 | 2508.3 ± 831 | -379.2 ± 815 | 118 ± 73 |
| Overweight | 8 (3%) | 203 (88%) | 21 (9%) | 2345.3 ± 617 | 2410.6 ± 767 | -73.6 ± 864 | 103 ± 59 |
| Obesity | 19 (8%) | 200 (86%) | 13 (6%) | 2548.4 ± 651 | 2452 ± 708 | 92 ± 911 | 96 ± 48 |
| TOTAL | 45(3%) | 1171 (81%) | 223 (15%) | 2221 ± 621 | 2484 ± 796 | -267 ± 859 | 128 ± 67 |

Values presented as n (%), or mean ± SD. *Energy expenditure = Basal metabolic rate (from Shofield equations) x physical activity level.
